# Healthcare provisions associated with multiple HIV‐related outcomes among adolescent girls and young women living with HIV in South Africa: a cross‐sectional study

**DOI:** 10.1002/jia2.26212

**Published:** 2024-02-08

**Authors:** Elona Toska, Siyanai Zhou, Christina A. Laurenzi, Wylene Saal, William Rudgard, Camille Wittesaele, Nontokozo Langwenya, Janina Jochim, Boladé Hamed Banougnin, Laurie Gulaid, Alice Armstrong, Gayle Sherman, Olanrewaju Edun, Lorraine Sherr, Lucie Cluver

**Affiliations:** ^1^ Centre for Social Science Research University of Cape Town Cape Town South Africa; ^2^ Department of Social Policy and Intervention University of Oxford Oxford UK; ^3^ Division of Social and Behavioural Sciences Faculty of Health Sciences University of Cape Town Cape Town South Africa; ^4^ Institute for Life Course Health Research Department of Global Health Faculty of Medicine and Health Sciences Stellenbosch University Cape Town South Africa; ^5^ Department of Infectious Disease Epidemiology London School of Hygiene & Tropical Medicine London UK; ^6^ UNICEF Eastern and Southern Africa Office (UNICEF‐ESARO) Nairobi Kenya; ^7^ National Institute for Communicable Diseases Johannesburg South Africa; ^8^ Department of Paediatrics and Child Health Faculty of Health Sciences University of the Witwatersrand Johannesburg South Africa; ^9^ MRC Centre for Global Infectious Disease Analysis School of Public Health Imperial College London London UK; ^10^ Institute for Global Health University College London London UK; ^11^ Department of Psychiatry and Mental Health University of Cape Town Cape Town South Africa

**Keywords:** adolescents, motherhood, adherence, treatment, health services, South Africa

## Abstract

**Introduction:**

Adolescent girls and young women (AGYW) living with HIV experience poor HIV outcomes and high rates of unintended pregnancy. Little is known about which healthcare provisions can optimize their HIV‐related outcomes, particularly among AGYW mothers.

**Methods:**

Eligible 12‐ to 24‐year‐old AGYW living with HIV from 61 health facilities in a South African district completed a survey in 2018–2019 (90% recruited). Analysing surveys and medical records from *n* = 774 participants, we investigated associations of multiple HIV‐related outcomes (past‐week adherence, consistent clinic attendance, uninterrupted treatment, no tuberculosis [TB] and viral suppression) with seven healthcare provisions: no antiretroviral therapy (ART) stockouts, kind and respectful providers, support groups, short travel time, short waiting time, confidentiality, and safe and affordable facilities. Further, we compared HIV‐related outcomes and healthcare provisions between mothers (*n* = 336) and nulliparous participants (*n* = 438). Analyses used multivariable regression models, accounting for multiple outcomes.

**Results:**

HIV‐related outcomes were poor, especially among mothers. In multivariable analyses, two healthcare provisions were “accelerators,” associated with multiple improved outcomes, with similar results among mothers. Safe and affordable facilities, and kind and respectful staff were associated with higher predicted probabilities of HIV‐related outcomes (*p*<0.001): past‐week adherence (62% when neither accelerator was reported to 87% with both accelerators reported), clinic attendance (71%−89%), uninterrupted ART treatment (57%−85%), no TB symptoms (49%−70%) and viral suppression (60%−77%).

**Conclusions:**

Accessible and adolescent‐responsive healthcare is critical to improving HIV‐related outcomes, reducing morbidity, mortality and onward HIV transmission among AGYW. Combining these provisions can maximize benefits, especially for AGYW mothers.

## 1 INTRODUCTION

Adolescent girls and young women (AGYW) aged 15–24 represented nearly one‐quarter of new HIV infections in 2022 in sub‐Saharan Africa [1]. In parallel, AGYW living with HIV in sub‐Saharan Africa continue to experience early pregnancy: nearly 30% of AGYW have had a child before age 20 [2, 3]. Recent multi‐country analyses of nationally representative Eastern and Southern Africa datasets identified strong associations between HIV prevalence and early motherhood, highlighting the importance of these overlapping vulnerabilities for HIV programming and maternal care [2, 4].

AGYW living with HIV experience multiple adverse HIV‐related health outcomes, including poorer adherence to antiretroviral therapy (ART), retention in care, clinic attendance and viral suppression compared to older women [5]. Enhancing their health and survival requires simultaneously improving multiple health outcomes. Until recently, no systematic reviews identified interventions that effectively improved multiple HIV‐related outcomes among adolescents living with HIV (ALHIV) [6, 7]. Recently, promising community‐based interventions have emerged, such as the peer‐facilitated psychosocial support for adolescents and young people living with HIV in Zimbabwe, and a livelihoods‐focused support package in Uganda [7, 8, 9].

However, no studies have identified which healthcare factors can improve HIV‐related outcomes among AGYW living with HIV, nor among mothers. The World Health Organization has set valuable and aspirational global standards for quality healthcare services for adolescents [10]. However, in over‐burdened, under‐resourced health systems, such as South Africa's, successfully achieving these standards at scale remains challenging. Studies with ALHIV have identified potential healthcare factors associated with improved adolescent HIV‐related outcomes: no medication/ART stockouts, kind and respectful healthcare providers, confidentiality, short travel time to facilities, short waiting time at facilities and accessible facilities [11, 12]. We need to understand which factors have the greatest impact on multiple HIV‐related outcomes in real‐world healthcare delivery—government primary healthcare settings within high HIV‐burden communities. Identifying healthcare provisions that act as “healthcare accelerators”—provisions that improve multiple health outcomes concurrently—can help us maximize healthcare investments, and guide provider training and facility improvements.

This study was co‐designed with the South African National Department of Health and UNICEF Eastern and Southern Africa Office, responding to a need to identify which healthcare provisions to prioritize for AGYW, including AGYW who are mothers, living with HIV [13]. This analysis investigates associations of seven healthcare provisions with five HIV‐related outcomes in a large study of AGYW living with HIV in South Africa. We conduct additional sub‐analyses focusing on AGYW living with HIV who are mothers, an understudied and priority population [4].

## 2 METHODS

### 2.1 Procedures and data

We analysed data from all AGYW living with HIV in the Mzantsi Wakho and HEY BABY studies in the Eastern Cape province of South Africa. All AGYW living with HIV, aged 12–24, from 52 government clinics and nine maternity obstetric units in a health district in South Africa's Eastern Cape Province were invited to participate in the study. Although conventionally AGYW refers to 15‐ to 24‐year‐olds, we included participants <15 years old given the importance of this growing cohort of very young mothers, and limited research on their outcomes. Over 90% of eligible participants were enrolled in the study in each facility type; 90.1% and 96%, respectively [14, 15], completed a survey on their multi‐dimensional experiences of health, HIV and healthcare experiences. HIV status was ascertained through medical records, including either a confirmed HIV‐positive test result, CD4 count or viral load (VL) at treatment initiation prior to the interview [16]. We interviewed both AGYW living with HIV mothers (participants who had had their first child before age 20, excluding *n* = 18 who had their first child >20) and nulliparous AGYW living with HIV (participants who have never given birth to a live child). A final sample of *n* = 774 AGYW living with HIV participated in the study from 2018 to 2019. Self‐reported questionnaires—using validated tools where available—were piloted with AGYW living with HIV, including AGYW who were mothers [17].

Participant medical records on VL, CD4 count and WHO staging were individually linked using unique study identifiers, following stringent data protection, consent and management protocols. Medical records data extracted from *n* = 67 health facilities were supplemented by routine laboratory test data from the National Health Laboratory Services (NHLS) data warehouse which archives all routinely collected public sector laboratory data from South Africa's National HIV Programme. Demographic information (name, surname, sex, date of birth, health facility location) for participants in the study accessing public sector HIV care were linked to laboratory test records in the NHLS and used to extract adolescents’ HIV VL records, following consent by participants and caregivers. Laboratory tests performed both within and outside of the Eastern Cape were accessed through national record linkages. Test record linkages in the data warehouse are achieved by a rule‐ and probabilistic matching‐based algorithm using patient demographics to assign multiple tests to a single patient [18]. In total, VL results were available for 60% (467/774) of participants included in this analysis. Following the merge, data were de‐identified before analyses. If multiple records were available for each participant, the record closest to the interview date (within a year) was included in this analysis.

Voluntary informed consent was obtained from adolescents and their caregivers when adolescents were under 18 years, following international and national guidelines, including data linkages approval. Ethical approvals were obtained from the Universities of Oxford (R48876/RE001,SSD/CUREC2/12–21) and Cape Town (HREC226/2017,CSSR2013/4,CSSR 2017/01), Eastern Cape Departments of Health and Basic Education, NHLS Academic Affairs and Research Management System (2019/08/07) and participating health and educational facilities. Participants received a certificate and a gift pack selected by the study's Teen Advisory Group, including toiletries for AGYW and their infants.

### 2.2 Measures

We measured five HIV‐related outcomes: (i) past‐week ART adherence; (ii) consistent clinic attendance; (iii) uninterrupted ART treatment; (iv) viral suppression; and (v) no tuberculosis (TB) symptomatology. Past‐week ART adherence was defined based on self‐report of currently taking ART and not having missed any doses in the past 7 days (including weekdays and weekends) [19]. Self‐reported consistent clinic attendance was measured as participants reporting missing none of their clinic appointments in the last year. Past‐year uninterrupted ART treatment was coded as 1 if participants self‐reported no treatment interruptions of 2+ days in a row, or 0 otherwise [20]. Viral suppression was defined as having a VL <1000 copies/ml at the most recent VL measurement up to 12 months following the interview date. Given very low rates of TB testing among ALHIV, no TB symptomology was measured using an algorithm based on the five most common pulmonary TB symptoms (i.e. dry cough for >2 weeks, weight loss, night sweats, chest pain, fever) [21]. Participants experiencing TB symptoms based on the algorithm were coded as 0, with no symptoms coded as 1. Self‐reported ART outcomes data can pose reliability and validity challenges, but in this study, longitudinal analyses indicated strong associations between the above self‐reported HIV‐related outcomes and VL data over time, for the subsample with available VL data [22].

Socio‐demographic factors included: *age* (grouped: <15, 15–19 and 20–24 years), *residence* (urban/rural, using the 2011 South African census); *housing* (informal/formal); *household poverty*, measured as missing one of the seven highest socially perceived necessities for adolescents (e.g. enough clothes), validated in South Africa; and *food insecurity*, measured by combining (1) participant did not have enough food for the entire week and (2) participants could not afford three daily meals at home. Participants who reported having at least one child <20 years old were coded as *AGYW mothers*.

Other HIV‐related factors included as control variables: *time on treatment*, measured as years since ART initiation (based on medical records or, when no records were available, self‐reported age at ART initiation). This was coded as *recently initiated* (0−3 years on treatment) versus all others. *Mode of HIV acquisition* (*recent/perinatal*) was computed via an algorithm based on the age of ART initiation, validated with self‐reported data such as the age of first sex, orphanhood cause and experiences of sexual assault [23].

Seven healthcare provisions were co‐identified with adolescent advisors during piloting based on a participatory activity to design the Dream Clinic [13]. All provisions were coded so that 1 = positive healthcare experiences and were measured in the past year. *No ART stockouts* were computed as a dichotomous variable if the participant reported experiencing no ART stockouts at the clinic (coded as 1) in the past year. *Kind and respectful providers* were measured based on adolescent satisfaction with the quality of care and “never in the past year” reporting either of the two negative clinic experiences: “Clinic staff got angry and scolded me because of how I take my pills,” and “Clinic staff got angry with me because I am having sex and shouted at me.” *Support group attendance* was measured using adolescent self‐report of attending a facility‐linked HIV support group in the past year. *Short travel time* and *short waiting time* were both measured as <60 minutes typically spent travelling to a health facility and time spent waiting to see a healthcare provider. *Confidentiality* was computed based on responses to adolescents’ response to the following question, linked to facility‐based services: “I felt that my information would be kept confidential (never, once or twice, several times, and most of the time).” Participants who reported “several times, and most of the time” to the question were coded as experiencing confidentiality. Given the importance of confidentiality in shaping adolescent healthcare access, we conducted sensitivity analyses with different cut‐off levels (never vs. any experiences of confidentiality); however, the results did not change considerably. *Safe and affordable facilities* were defined based on adolescents reporting whether they could *afford* to get to the doctor, clinic or hospital, and whether they *felt safe* at the clinic/hospital in the past year.

### 2.3 Analysis

Analyses were conducted in Stata Version 17.0. First, descriptive statistics for all variables (HIV‐related factors, socio‐demographic, HIV‐related outcomes and healthcare provisions) were computed comparing AGYW mothers living with HIV (*n* = 336) to nulliparous AGYW living with HIV (*n* = 438) using Chi‐square tests. Supplementary analyses explored differences between participants with matched medical records. Matched participants (*n* = 467) were, on average, a few months younger than those with no VL data (*n* = 307), more likely to live in rural settings, less likely to live in a poor household and more likely to have acquired HIV recently through presumed sexual exposure (Table S1). We adjusted for these factors in the consequent analysis.

Analysis for the viral suppression outcome was conducted in a sub‐sample of participants with available data (*n* = 467), with *n* = 193 AGYW mothers living with HIV and *n* = 274 AGYW living with HIV matched. Second, multicollinearity checks for the five outcomes of interest were conducted using tetrachoric correlations, since all the outcomes were categorical. All correlations were weak‐moderate (<0.7), except for the correlation between past‐week adherence, consistent clinic attendance and uninterrupted ART treatment. To adjust for these correlations, *p*‐*values* for each model were adjusted for multiple outcome testing using the Benjamini–Hochberg approach [24]. Third, associations between each HIV‐related outcome and healthcare provisions were explored using multivariate logistic regression models, controlling for socio‐demographic characteristics, motherhood and HIV‐related factors. We used multivariate logistic regression analysis to examine associations between the above seven *healthcare provisions* and five HIV‐related outcomes among (i) AGYW living with HIV, and (ii) disaggregated by motherhood. We followed an empirical approach to identify “development accelerators”—provisions associated with two or more outcomes simultaneously, refined and used with multiple observational datasets available in Open Science Framework [25, 26].

In the final step, healthcare provisions associated with two or more HIV‐related outcomes of the above regressions were considered “healthcare accelerators” and included in a model controlling for the above HIV and socio‐demographic covariates. To explore associations between healthcare accelerators and HIV‐related outcomes for AGYW mothers, we conducted sub‐group analyses by motherhood status, quantifying associations between accessing the seven healthcare provisions on the five HIV‐related outcomes for nulliparous and mothering AGYW living with HIV in two separate models. Finally, predicted probabilities to model the impact of healthcare provisions—alone and in combination—on each HIV‐related outcome were computed only for healthcare accelerators, for the full sample and the two subgroups by motherhood status: mothers and nulliparous AGYW living with HIV, separately.

## 3 RESULTS

### 3.1 Sample characteristics, HIV‐related factors and healthcare provisions

Of all AGYW living with HIV (*N* = 774), 43.4% were mothers. Socio‐demographic characteristics and HIV‐related outcomes by motherhood are shown in Table 1. AGYW mothers were more likely to be older, have recently acquired HIV and recently initiated treatment (<3 years). They were also more likely to report past‐week food insecurity, living in informal housing and poorer households. Regarding HIV‐related outcomes, AGYW mothers living with HIV were more likely to report lower rates of past‐week ART adherence (*p* = 0.013), consistent clinic attendance (*p* = 0.007), uninterrupted ART treatment (*p*<0.001) and TB symptoms (*p*<0.001). About 60% of all participants had a VL test and there was no difference in available VL data by motherhood status (Table S1). A lower proportion of VL data was available among participants with recently acquired HIV and who lived in poorer households. Among those with VL results, viral suppression rates were lower among mothers compared to nulliparous AGYW living with HIV (*p*<0.001). AGYW mothers living with HIV were less likely to report receiving four of the seven healthcare provisions—adolescent‐responsive services (*p* = 0.018), support group attendance (*p* = 0.005), accessible care (*p* = 0.033) and short waiting times (*p*<0.001)—compared to AGYW living with HIV.

**Table 1:** Socio‐demographic characteristics, HIV‐related outcomes and healthcare services by motherhood status

Factor (*n*, %)	Total	AGYW mothers living with HIV	AGYW living with HIV	
Factors	(*N*=774)	(*N*=336)	(*N*=438)	*p*‐value
**Socio‐demographic characteristics**				
Age group				**<0.001**
1. 12−14	152 (19.2%)	2 (0.6%)	150 (34.2%)	
2. 15−19	388 (49.0%)	161 (47.9%)	227 (51.8%)	
3. 20−24	234 (30.2%)	173 (51.5%)	61 (13.9%)	
Rural	190(24.5%)	84 (25.1%)	106 (24.3%)	0.790
Informal housing	143 (18.5%)	80 (24.7%)	63 (14.4%)	**<0.001**
Poverty	581 (75.1%)	275 (81.8%)	306 (69.9%)	**<0.001**
Recent HIV acquisition	360 (46.5%)	284 (87.9%)	76 (17.6%)	**<0.001**
Food insecurity	219 (28.3%)	115 (34.2%)	104 (23.7%)	**0.001**
Time on treatment (<3 years)	209 (27.0%)	160 (51.3%)	49 (11.3%)	**<0.001**
**HIV‐related outcomes**				
Past‐week ART adherence^a^	604 (78.0%)	248 (73.8%)	356 (81.3%)	**0.013**
Consistent clinic attendance^a^	626 (80.9%)	257 (76.5%)	369 (84.2%)	**0.007**
Uninterrupted ART treatment^a^	582 (75.2%)	226 (67.3%)	356 (81.3%)	**<0.001**
TB free (no TB symptoms)^a^	503 (65.0%)	293 (87.2%)	210 (47.9%)	**<0.001**
VL suppression (<1000 copies/ml)^b^	341 (73.0%)	124 (64.2%)	217 (79.2%)	**<0.001**
**Healthcare service provisions**				
No medication stock outs	744 (96.1%)	323 (96.1%)	421 (96.1%)	0.990
Kind and respectful staff	660 (85.3%)	275 (81.8%)	385 (87.9%)	**0.018**
Support group	49 (6.3%)	12 (3.7%)	37 (8.9%)	**0.005**
Short travel time (<1 hour)	734 (94.8%)	317 (94.3%)	417 (95.2%)	0.590
Short waiting time (<1 hour)	327 (42.2%)	118 (35.1%)	209 (47.7%)	**<0.001**
Confidentiality	434 (56.1%)	186 (55.4%)	248 (56.6%)	0.730
Safe and affordable care	685 (88.5%)	288 (85.7%)	397 (90.6%)	**0.033**

^a^ Self‐reported measures.

^b^ Analysis for the viral suppression outcome were conducted in a sub‐sample of participants with available data (*n* = 467), with *n* = 193 AGYW mothers living with HIV and *n* = 274 AGYW living with HIV matched.

### 3.2 Multivariable associations between healthcare provisions and HIV‐related outcomes

In multivariable analyses, of the seven healthcare provisions, three were associated with any HIV‐related outcomes (Table 2). No ART stockouts were associated with one outcome: uninterrupted ART treatment (aOR: 2.56, 95% CI 1.12−5.81, *p* = 0.025). Kind and respectful providers, and safe and affordable clinics, were associated with improvements in three out of five included HIV‐related outcomes. Adolescent‐responsive services were associated with higher odds of past‐week ART adherence (aOR: 2.89, 95% CI 1.75−4.77, *p*<0.001), consistent clinic attendance (aOR: 1.72, 95% CI 1.00−2.97, *p* = 0.051), uninterrupted ART treatment (aOR: 2.26, 95% CI 1.39−3.67, *p* = 0.001) and viral suppression (aOR: 2.59, 95% CI 1.42−4.74, *p* = 0.002). Accessible care was associated with higher odds of consistent clinic attendance (aOR: 1.87, 95% CI 1.01−3.44, *p* = 0.045), uninterrupted ART treatment (aOR: 1.95, 95% CI 1.11−3.42, *p* = 0.019) and no TB symptoms (aOR: 1.82, 95% CI 1.03−3.20 *p* = 0.038). Accessing support groups, short travel and waiting time (<1 hour for each), and confidential healthcare services were not associated with any of the HIV‐related outcomes, when controlling for covariates.

**Table 2:** Summary of associations between healthcare provisions and HIV‐related outcomes (*N* = 774)

	Past‐week adherence^a^	Consistent clinic attendance^a^	Uninterrupted ART treatment^a^	No TB symptoms^a^	Viral suppression (<1000 copies/ml)^b^
**Healthcare factors**	**aOR (95% CI)** ***p*‐value** ^c^				
No ART stock outs	0.84 (0.29−2.45) 0.746	0.90 (0.29−2.76) 0.850	1.48 (0.60−3.66) 0.401	0.97 (0.40−2.32) 0.943	0.99 (0.30−3.22) 0.986
Kind and respectful providers	**2.89 (1.75**−**4.77)** **<0.001**	**1.72 (1.00**−**2.97)** **0.051**	**2.26 (1.39**−**3.67)** **0.001**	1.34 (0.81−2.23) 0.256	**2.59 (1.42**−**4.74)** **0.002**
Support group	1.91 (0.71−5.17) 0.202	0.85 (0.36−2.04) 0.720	0.93 (0.42−2.08) 0.864	0.70 (0.36−1.36) 0.292	1.39 (0.57−3.35) 0.467
Short travel time (< 1 hour)	0.98 (0.63−1.51) 0.912	0.93 (0.58−1.48) 0.763	1.02 (0.67−1.55) 0.928	1.24 (0.87−1.79) 0.238	0.73 (0.46−1.18) 0.200
Short waiting time (<1 hour)	1.05 (0.43−2.52) 0.919	0.66 (0.24−1.83) 0.423	1.16 (0.52−2.59) 0.709	1.00 (0.47−2.11) 0.991	1.30 (0.49−3.47) 0.604
Confidentiality	0.91 (0.59−1.41) 0.676	1.05 (0.66−1.66) 0.834	0.93 (0.62−1.41) 0.746	0.72 (0.50−1.03) 0.076	0.80 (0.50−1.28) 0.353
Safe and affordable facilities	1.42 (0.78−2.60) 0.248	**1.87 (1.01**−**3.44)** **0.045**	**1.95 (1.11**−**3.42)** **0.019**	**1.82 (1.03**−**3.20)** **0.038**	0.85 (0.41−1.78) 0.673
**Covariates**					
Age (groups)	(0.91−1.10) 0.991	1.02 (0.92−1.13) 0.705	0.99 (0.90−1.09) 0.903	1.03 (0.96−1.11) 0.404	**0.89 (0.80**−**0.99)** **0.035**
Rural residence	**1.81 (1.05**−**3.12)** **0.032**	1.46 (0.83−2.57) 0.187	1.13 (0.70−1.84) 0.620	0.94 (0.62−1.43) 0.765	1.21 (0.71−2.06) 0.483
Informal housing	0.74 (0.45−1.22) 0.238	0.85 (0.50−1.44) 0.540	0.67 (0.42−1.09) 0.105	0.73 (0.46−1.15) 0.176	1.02 (0.58−1.79) 0.934
Poverty	0.65 (0.37−1.12) 0.119	**0.52 (0.28**−**0.96)** **0.038**	0.77 (0.46−1.27) 0.303	0.97 (0.65−1.45) 0.889	0.85 (0.50−1.46) 0.564
Food insecurity	**0.62 (0.39**−**0.97)** **0.037**	0.83 (0.51−1.36) 0.462	0.90 (0.58−1.41) 0.656	0.93 (0.62−1.40) 0.726	**0.47 (0.28**−**0.77)** **0.003**
Time on treatment	**1.78 (1.04**−**3.07)** **0.037**	1.11 (0.64−1.93) 0.712	**2.10 (1.26**−**3.50)** **0.005**	**2.66 (1.59**−**4.47)** **<0.001**	1.21 (0.66−2.23) 0.533
Mode of HIV acquisition	0.53 (0.27−1.04) 0.066	0.52 (0.25−1.09) 0.083	**0.35 (0.18**−**0.66)** **0.001**	0.96 (0.56−1.68) 0.899	1.52 (0.73−3.18) 0.267
Motherhood	0.93 (0.50−1.74) 0.816	0.91 (0.46−1.79) 0.788	0.80 (0.45−1.45) 0.469	**5.57 (3.16**−**9.81)** **<0.001**	0.58 (0.28−1.20) 0.140

Abbreviations: aOR, adjusted odds ratio; 95% CI, confidence interval.

^a^ Self‐reported measures.

^b^ Analyses for the viral suppression outcome were conducted in a sub‐sample of participants with available data (*n* = 467), with *n* = 193 AGYW mothers living with HIV and *n* = 274 AGYW living with HIV matched.

^c^ Adjusted for multiple testing using Benjamini–Hochberg approach with a false discovery rate (FDR) of 5%.

### 3.3 Healthcare accelerators for AGYW living with HIV: individual and combined effect of healthcare provisions and HIV‐related outcomes

To investigate the individual and combined effect of accessing only the healthcare accelerators identified above with improvements in multiple HIV‐related outcomes, we modelled adjusted probabilities for each of the five HIV‐related outcomes, using four scenarios: (1) no access to either healthcare accelerator; (2) access to kind and respectful providers; (3) access to safe and affordable facilities; and (4) access to both (Table 3). Adjusted probabilities of all five HIV‐related outcomes were higher for the scenario in which kind and respectful providers and safe and affordable facilities were experienced together, compared to a scenario of neither. The adjusted probabilities when comparing the scenario with neither adolescent‐responsive services nor accessible care (i.e. no healthcare accelerator) to the scenario with a combination of both healthcare accelerators increased from 62% to 87% (+25 percentage point [pp] increase) for past‐week ART adherence, from 71% to 89% (+18 pp) for consistent clinic attendance, from 57% to 85% (+28 pp) for uninterrupted ART treatment, from 49% to 70% (+21 pp) for no TB symptoms and from 60% to 77% (+17 pp) for viral suppression.

**Table 3:** Adjusted predicted probabilities of HIV‐related outcomes comparing different combinations of healthcare accelerators (*n* = 774)

	Predicted probabilities of each outcome (95% CI)				
Healthcare factors	Past‐week adherence	Consistent clinic attendance	Uninterrupted ART treatment	No TB symptoms	Viral suppression (<1000 copies/ml)^a^
(1) No healthcare accelerators	62% (47%−77%)	71% (58%−85%)	57% (42%−72%)	49% (34%−65%)	60% (42%−79%)
(2) Safe and affordable facilities	70% (61%−80%)	82% (75%−90%)	72% (63%−81%)	64% (53%−75%)	57% (43%−70%)
(3) Kind and respectful providers	83% (74%−91%)	81% (72%−90%)	75% (64%−85%)	56% (43%−70%)	80% (68%−91%)
(4) Both healthcare accelerators	87% (84%−90%)	89% (86%−92%)	85% (82%−88%)	70% (66%−75%)	77% (73%−82%)

^a^ Analyses for the viral suppression outcome were conducted in a sub‐sample of participants with available data (*n* = 467).

**Figure 1:**
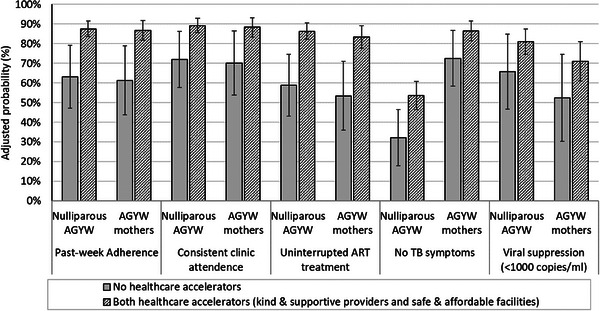
Associations between healthcare accelerators and HIV‐related outcomes by motherhood.

In a stratified analysis, the effect of healthcare accelerators for AGYW all living with HIV by motherhood status differed on only TB symptomology: mothers were less likely to experience TB symptoms. However, accessing both healthcare accelerators compared to accessing neither had a greater effect for nulliparous AGYW than mothers: the adjusted probability of no TB symptoms increased from 32% to 54% (+22 pp) for nulliparous AGYW living with HIV compared to an increase from72% to 87% (+15 pp) among AGYW who were mothers (Figure 1).

## 4 DISCUSSION

AGYW are at considerable risk of mortality and morbidity due to HIV‐related illness [27]. Supporting them to survive and thrive is an urgent public health and human rights concern [28, 29]. This paper explored healthcare provisions associated with improved HIV‐related outcomes among AGYW living with HIV, including AGYW who are mothers as a priority group. First, we investigated rates of HIV‐related outcomes among our sample of *n* = 774 AGYW living with HIV by motherhood. A high proportion of study participants reported sub‐optimal HIV‐related outcomes, similar to other studies from the region [30]. Differences in TB symptoms by motherhood status should be investigated in cohort studies with medical TB records.

Three healthcare provisions were associated with improvements in at least one HIV‐related outcome: no medication stockouts, kind and respectful providers, and safe and affordable facilities. Fully stocked facilities were associated with uninterrupted ART treatment, highlighting the need for continued access to life‐saving medication, echoing findings with younger ALHIV in South Africa [11]. This provision remains tenuous even as the COVID‐19 pandemic, during which many supply chains were interrupted, has subsided [31]. Addressing medication/ART stockouts promptly is critical to keeping AGYW living with HIV alive. Advocacy and support for providers is needed to help them report medication/ART stockout rather than informally manage them [12].

The other two healthcare provisions were each associated with improvements across multiple HIV‐related outcomes for AGYW living with HIV, meeting the study's criteria of “healthcare accelerators.” Kind and respectful providers were associated with improvements in four of the five HIV‐related outcomes: past‐week adherence, consistent clinic attendance, uninterrupted ART treatment and viral suppression. These findings support prior studies showing associations between respectful healthcare providers and retention in HIV care in younger ALHIV [11, 32]. Our findings further demonstrate improvements across multiple HIV‐related outcomes among AGYW living with HIV, including among mothers.

The second healthcare accelerator, reporting safe and affordable facilities, was associated with improvements in clinic attendance, uninterrupted treatment and TB symptoms. This finding supports and expands prior evidence that has found associations between affordable transport and retention in care among younger adolescents [11], and evidence from a randomized trial in Uganda showing positive impacts of an economic intervention on viral suppression [9]. New models of HIV care delivery, such as differentiated service delivery, peer‐ and community‐based care and mobile clinics, may increase acceptability, affordability and safety for AGYW [33, 34, 35, 36], and be important steps to improving their HIV outcomes.

AGYW living with HIV who were mothers reported consistently poorer adherence and more ART treatment interruptions than nulliparous AGYW. The same two healthcare provisions were associated with improved outcomes for AGYW living with HIV independent of motherhood status: kind and respectful providers and safe and affordable facilities. However, AGYW mothers reported lower levels of access to these healthcare provisions than other nulliparous AGYW living with HIV. This finding suggests that all adolescent girls living with HIV will benefit from the same healthcare provisions, but that additional efforts may be needed to ensure that they reach AGYW who are mothers [35].

Importantly, the two healthcare accelerators identified in this study are similar to healthcare factors that improve outcomes among all adolescents [11, 37]. This finding suggests that strengthening healthcare systems and quality of care for adolescents, in general, may benefit the highest‐risk groups, such as AGYW living with HIV, including young mothers—an important finding given the higher likelihood of poorer HIV‐related outcomes among AGYW living with HIV who are mothers. AGYW living with HIV, particularly mothers, who experienced both kind and respectful staff and safe and affordable facilities were more likely to report higher rates of multiple HIV‐related outcomes, including viral suppression, than those accessing each individual healthcare accelerator. Future analyses should investigate whether combinations have additive or synergistic relationships using longitudinal and experimental designs.

This study has several limitations. First, the data were collected through a cross‐sectional survey, and so all findings must be interpreted with caution. However, we were careful in selecting variables and their reporting or recall periods to minimize the risk of reverse causality. Second, HIV‐related measures were self‐reported, as VL data were only available for a part of the sample, due to limited VL monitoring and data quality in the study catchment area. Although self‐reported adherence and related measures have poor reliability and validity, longitudinal analyses of these measures showed a strong association with VL data over time [22]. Efforts are underway to improve data infrastructure, which would allow for longitudinal analyses to explore the long‐term outcomes of these healthcare provisions. Participants living in poorer households and who had recently acquired HIV were less likely to have matched VL measures, so we accounted for these two variables in multivariate analysis. Third, the data were collected just before the COVID‐19 pandemic, and the pandemic may have shifted some of the dynamics we observed in these data, especially access to VL testing. Additional research is needed to document the post‐pandemic experiences of AGYW living with HIV, particularly mothers. Fourth, while we accounted for covariates, there may be unmeasured factors, including household and community services and support. However, we conducted multivariate analyses controlling for key covariates and accounted for multiple outcome testing. Finally, this study was conducted in the Eastern Cape province of South Africa, and its findings may not be generalizable to other settings. However, the challenges facing our participants, including HIV risk, poverty and early motherhood, are similar to those in other parts of sub‐Saharan Africa.

## 5 CONCLUSIONS

Despite these limitations, our study has important implications for supporting a large and growing cohort of AGYW living with HIV in the region. In light of increasing rates of adolescent motherhood [28], we urgently need research on which interventions are effective, in supporting healthcare providers to deliver adolescent‐sensitive services, reduce morbidity and mortality among AGYW living with HIV. Reaching AGYW living with HIV, particularly mothers, with safe, affordable HIV and health services requires kind, responsive healthcare service provision, and care that respects their dignity and supports them reach their full potential.

## AUTHORS’ CONTRIBUTIONS

ET and LC conceptualized the overall study. ET designed the analyses for this manuscript, which was conducted by SZ and ET, with support from WR and BHB. CW, NL and JJ were involved in study realization, data preparation and together with LS, WS, CAL, AA and LG contributed to data interpretation and writing. SZ and OE led the NHLS data merge with GS. All authors have reviewed the manuscript.

## COMPETING INTERESTS

The authors declare no competing interests.

## FUNDING

This study was funded by the UK Medical Research Council (MRC) and the UK Department for International Development (DFID) under the MRC/DFID Concordat agreement, and by the Department of Health Social Care (DHSC) through its National Institutes of Health Research (NIHR) [MR/R022372/1]; Evidence for HIV Prevention in Southern Africa (EHPSA), a UK aid programme managed by Mott MacDonald; Janssen Pharmaceutica N.V., part of the Janssen Pharmaceutical Companies of Johnson & Johnson; the John Fell Fund [161/033]; the Philip Leverhulme Trust [PLP‐2014‐095]; UCL's HelpAge funding; the European Research Council (ERC) under the European Union's Horizon 2020 research and innovation programme (n° 771468); the UKRI GCRF Accelerating Achievement for Africa's Adolescents (Accelerate) Hub (Grant Ref: ES/S008101/1); the Fogarty International Center, National Institute on Mental Health, National Institutes of Health under Award Number (K43TW011434 and D43TW011308); a CIPHER grant from International AIDS Society [155‐Hod; 2018/625‐TOS]; Research England [0005218]; the University of Oxford's ESRC Impact Acceleration Account [K1311‐KEA‐004]; a partnership and collaboration agreement with UNICEF ESARO made possible through the 2gether 4SRHR programme funded by the Government of Sweden; the Oak Foundation [OFIL‐20‐057]; the Nuffield Foundation; the Wellspring Philanthropic Fund [16204].

## DISCLAIMER

The content is solely the responsibility of the authors and does not represent the official views of the National Institutes of Health, the Nuffield Foundation or the official policies of the International AIDS Society.

## Supporting information

Supporting Information

## Data Availability

The data that support the findings of this study are available from the corresponding author upon reasonable request.
