# Supplementary material for: Healthcare provisions associated with multiple HIV‐related outcomes among adolescent girls and young women living with HIV in South Africa: a cross‐sectional study
Source: J Int AIDS Soc. 2024 Feb 8;27(2):e26212. doi: 10.1002/jia2.26212 (PMC10853575; doi:10.1002/jia2.26212)
Supplement: Supplementary file 1 — Supporting Information [file JIA2-27-e26212-s001.docx]

JIAS-2023-04-0171.R2

**Healthcare provisions associated with multiple HIV-related outcomes among adolescent girls and young women living with HIV in South Africa: a cross-sectional study**

**Running head:** Healthcare accelerators for AGYW living with HIV

**Supplementary Table**

**Table S1: Socio-demographic factors by available viral load measurements (N=774)**

|  | **Total (N=774)** | **VL matched**  **(N=467)** | **VL missing**  **(N=307)** |  |
| --- | --- | --- | --- | --- |
| **Socio-demographics** | **N (%)** | **N (%)** | **N (%)** | **p-value** |
| Age (years)-*Mean (SD)* | 17.63 (3.07) | 17.34 (3.03) | 17.91 (3.10) | **0.011** |
| Rural residence | 190 (24.5%) | 127 (27%) | 63 (21%) | **0.039** |
| Informal housing | 143 (18.5%) | 89 (19%) | 54 (18%) | 0.630 |
| Poverty | 581 (75.1%) | 336 (72%) | 245 (80%) | **0.013** |
| Recent HIV acquisition | 173 (22.4%) | 91 (27%) | 82 (38%) | **0.005** |
| Food insecurity | 219 (28.3%) | 128 (27%) | 91 (30%) | 0.500 |
| Time on treatment (<3 years) | 209 (27%) | 122 (27%) | 87 (30%) | 0.270 |
| Motherhood | 336 (43.4%) | 193 (41%) | 143 (47%) | 0.150 |
